# Effect of interprofessional and intraprofessional clinical collaboration on patient related outcomes in multimorbid older patients – a retrospective cohort study on the Intensive Collaboration Ward

**DOI:** 10.1186/s12877-023-04232-2

**Published:** 2023-08-26

**Authors:** Simon T. de Gans, Gerdinique C. Maessen, Marjolein H. J. van de Pol, Marjan J. van Apeldoorn, Margot A. L. van Ingen-Stokbroekx, Niels van der Sloot, Carolina J. P. W. Keijsers, Babette C. van der Zwaard

**Affiliations:** 1 Jeroen Bosch Hospital, Jeroen Bosch Academy, PO BOX 90153, 5200 ME ‘S-Hertogenbosch, the Netherlands; 2 University Medical Center Utrecht, CRU + Master Program, Utrecht, the Netherlands; 3 Department of Primary and Community Care, Radboud University Medical Center, Nijmegen, the Netherlands; 4 Department of Internal Medicine, Jeroen Bosch Hospital, ‘S-Hertogenbosch, the Netherlands; 5 Jeroen Bosch Hospital, Intensive Collaboration Ward, ‘S-Hertogenbosch, the Netherlands; 6 Department of Pulmonary Medicine, Jeroen Bosch Hospital, ‘S-Hertogenbosch, the Netherlands; 7 Department of Geriatric Medicine, Department of Clinical Pharmacology, Jeroen Bosch Hospital, ‘S-Hertogenbosch, the Netherlands; 8 Department of Orthopedics, Jeroen Bosch Hospital, ‘S-Hertogenbosch, the Netherlands

**Keywords:** Interprofessional, Intraprofessional, Collaboration, Collaborative practice, Multimorbidity, Efficacy

## Abstract

**Background:**

The management and care of older patients with multiple health problems is demanding and complex. Interprofessional and intraprofessional collaboration has the potential to improve both the efficiency and the quality of care for these patients. However, it has proven difficult to demonstrate the efficacy of this approach in terms of objective patient-related outcomes. Recently, a care model with interprofessional and intraprofessional care was started, the Intensive Collaboration Ward (ICW). This ward combines *inter*professional care and *intra*professional care for older patients with multiple health problems. The aim of this study was to evaluate the effects of ICW care in older patients with multiple health problems.

**Methods:**

This retrospective cohort study evaluated the effects on patients outcomes. This was done by comparing patients of the new model, the ICW (ICW group), to a historical cohort of comparable patients who would have been eligible for the ICW (control group). Outcomes were medical consultations, allied health professional consultations, radiological procedures, waiting time for radiological procedures, change in primary treating specialty, length of hospital stay, readmission rate, and mortality rate. Linear and logistic regression analyses were performed, adjusted for baseline differences.

**Results:**

The ICW group required significantly fewer medical consultations than the control group. Calls to specialists from the emergency room decreased significantly, but there was no change in in-person consultations on the ER. 51% of control patients had ≥ 1 in-hospital consultation compared to 21% of ICW patients (*p* < 0.05). Patients in the ICW group received significantly more consultations with allied health professionals and more often had a change in primary treating specialty.

**Conclusions:**

Interprofessional and intraprofessional clinical collaboration on the ICW reduced in-hospital consultations and increased allied health professionals’ consultations. This approach may decrease fragmentation of care and provide more integrated, efficient and patient centered care. This may improve the overall care of older patients with multiple health problems.

## Background

Life expectancyis increasing and this increases the demand for health services, because of increased age-related multimorbidity [1, 2]. Health care utilization is high among patients with multimorbidity [3–5], multimorbidity is defined by the WHO as the coexistence of two or more health conditions in the same individual [6]. Such patients are at risk of receiving fragmented care, which leads to more emergency department visits [7], preventable hospitalizations [8], and higher costs [4, 5]. There is an urgent need to improve the efficiency and quality of care for older patients with multimorbidity, which may necessitate a change in how hospital care is provided; for example, the WHO advises interprofessional collaborative practice [9].

Interprofessional collaboration has the potential to improve the care of older patients with multimorbidity, making more efficient use of resources. Many interprofessional care models have been proposed, and although most clinical care workers believe in their efficacy [10, 11], the few studies investigating this have failed to detect major improvements in objective patient-related outcomes [12–14]. The more intensive collaboration models have yielded better results, reducing the length of stay and in-hospital mortality [15]. An example of such an intensive collaboration model is the Intensive Collaboration Ward (ICW), which was set up in the Jeroen Bosch Hospital in the Netherlands to provide combined interprofessional and intraprofessional care for older patients with multimorbidity. Interprofessional collaboration is defined as healthcare professionals from different professions working together, e.g. nurse and physical therapist. Intraprofessional collaboration is defined as healthcare professionals from different disciplines working together, e.g. a cardiologist and a pulmonologist.

The ICW has been shown to be effective in decreasing the length of stay and number of in-hospital consultations compared with regular wards [16]. However, some efficacy parameters still need to be investigated. Therefore, the aim of this study is to assess the efficacy of health care provided on an ICW, expressed as the number of medical consultations in the emergency room (ER) and on the ward, the number of radiological procedures, waiting time for radiological procedures, change in primary treating specialty, length of hospital stay, readmission rate, and mortality rate.

## Methods

### Study design

This retrospective cohort study evaluated the effects of combined *inter*professional (healthcare professionals from different professions working together, e.g. nurse and physical therapist) and *intra*professional (healthcare professionals from different disciplines working together, e.g. a cardiologist and a pulmonologist) care on the ICW on the health outcomes of patients with multimorbidity.

### Intensive Collaboration Ward (ICW)

**Fig. 1:**
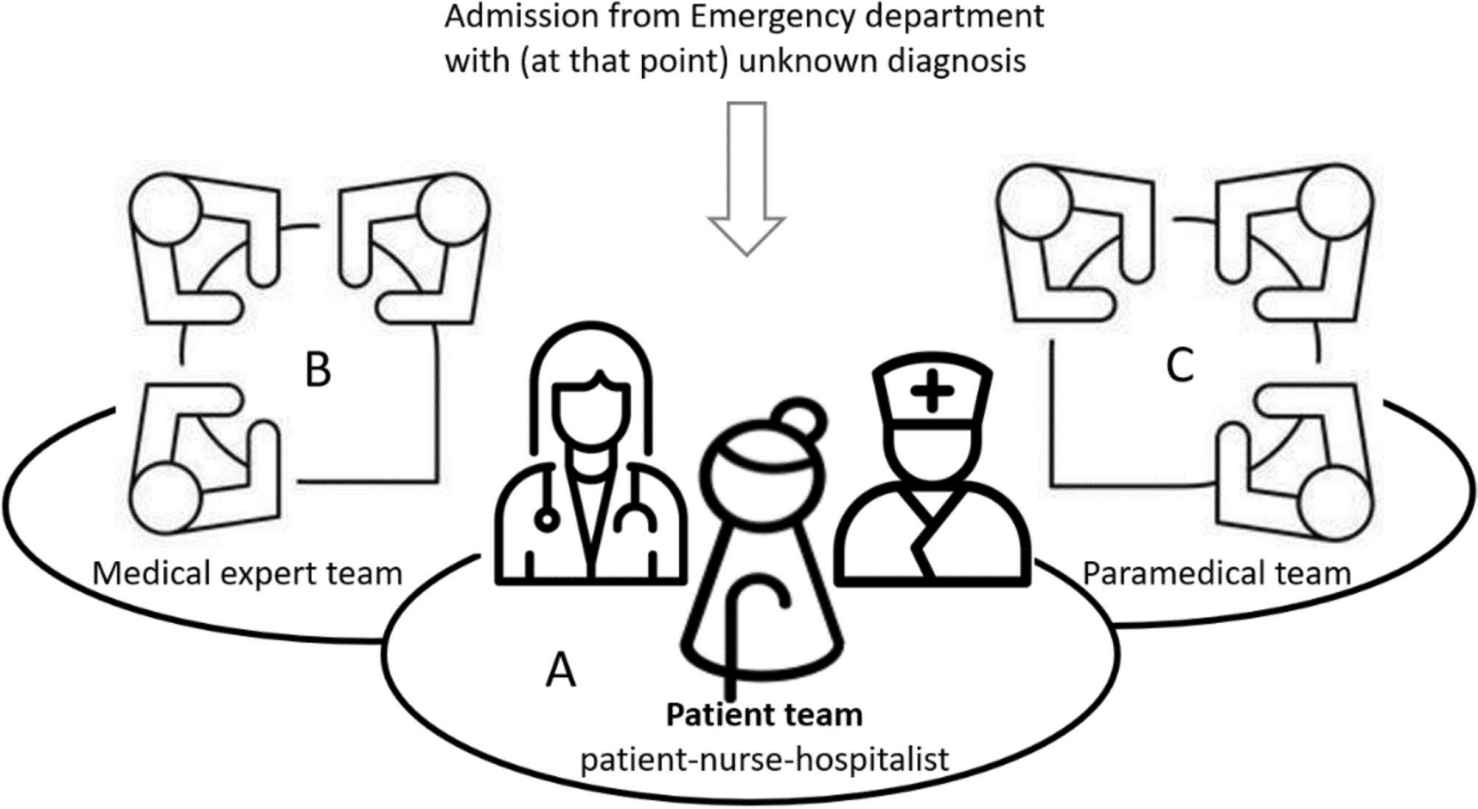
The operating procedures on the intensive collaboration ward The patient team, consisting of the patient, nurse and hospitalist, is central. The nurse and the hospitalist are the contacts for the patient and their family. The medical expert team consists of the hospitalist, and a geriatrician, internist, pulmonologist and cardiologist and are present at the Treatment Team Meeting every morning. The paramedical team consist of the hospitalist and nurse, and a psychical therapist, dietitian, speech therapist, occupational therapist, and liaison nurse. They come together 3 times a week. All teams work together to provide the best patient care for the older multimorbid patient

The ICW was set up to provide *inter*professional and *intra*professional care for older patients with multimorbidity. To care for these complex patients the ICW has several operating procedures, which have previously been described by de Gans et al. [16] The operating procedures are visualized in Fig. 1. The first principle is that ICW patients have one coordinating physician: the hospitalist. The hospitalist is a generalist who is specifically trained to evaluate the entirety of a patients’ health problems [17, 18]. A hospitalist is present 6 days a week, meaning the patient primarily sees one doctor on the ward. Second, there is a nursing team consisting of nurses from all involved specialties assuring a diverse background. The nurse and hospitalist work closely together and are the persons of contact for the patient and their family. Third, there is a Treatment Team Meeting (TTM) every morning at 9 am Monday to Saturday to represent the medical perspective of care. In this TTM each patient’s values and believes are introduced by the hospitalist as a starting point for the meeting. Subsequently, the patient is evaluated by the hospitalist together with a cardiologist, geriatrician, internist, and pulmonologist. The conversation is centered around the patient’s story. The medical specialists combine their expertise and all visions come together to collectively provide tailormade solutions for the patient. Fourth, the nurse and hospitalist meet three times a week with a team of allied health professionals to portray other aspects of the patients’ health. The involved allied health professionals are specifically assigned to the ICW and are a physical therapist, dietitian, speech therapist, occupational therapist, and liaison nurse. The ICW is an example of combined interprofessional and intraprofessional collaboration since these professionals work together, and regularly come together and negotiate to provide an integral solution for the patient. This is different from multidisciplinary or multiprofessional teamwork where professionals work parallel to each other and not necessarily negotiate an integral solution [19, 20]. In clinical practice, the definitions of multidisciplinary and interprofessional are often used inconsistently. For example, multidisciplinary teams in ICU also negotiate to provide an integral solution for the patient and could be described as interprofessional. For the purposes of this paper, the definitions used are as described in the literature.

**Fig. 2:**
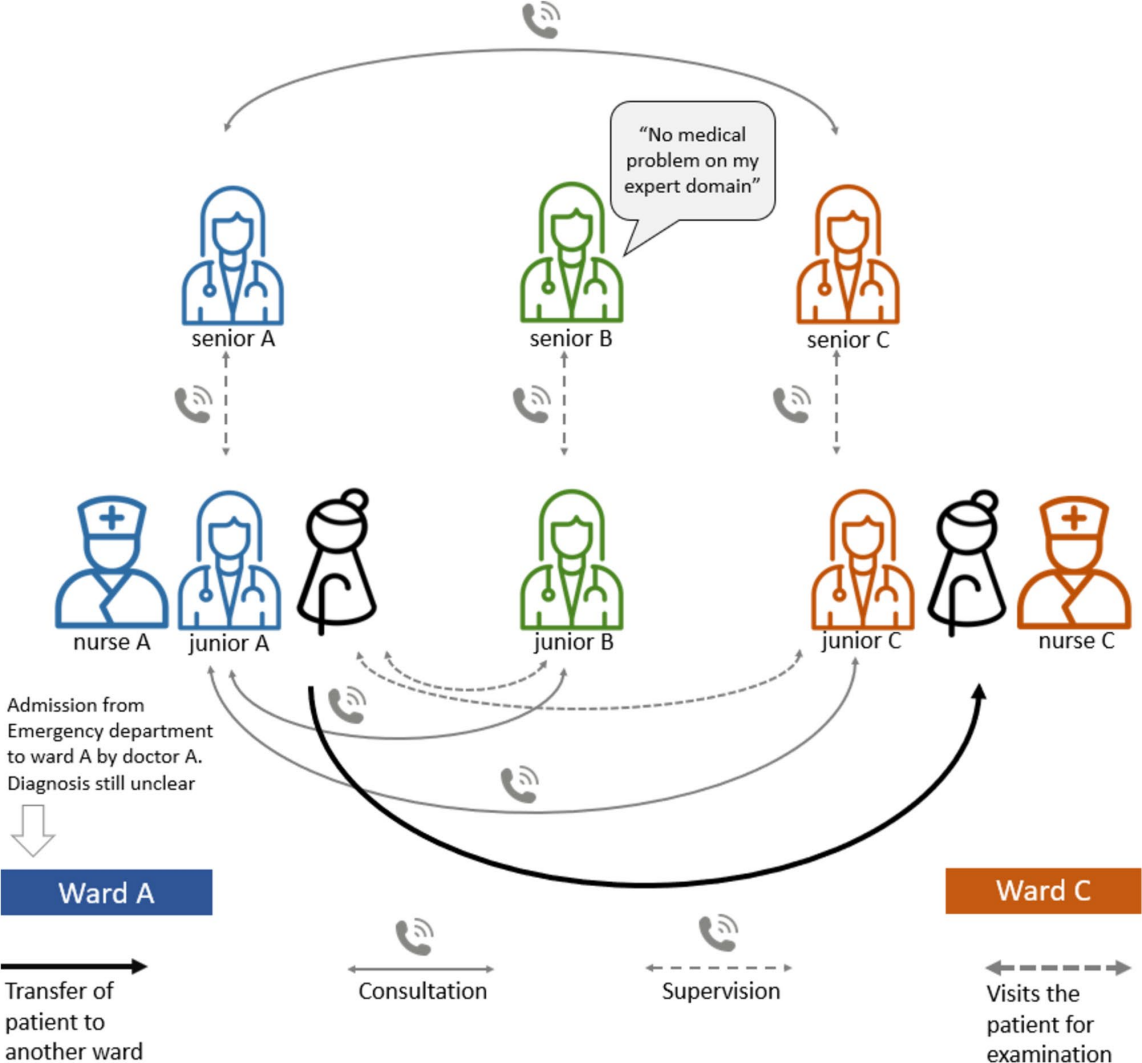
The operating procedures on a regular care ward As shown above, organization of care on a regular care ward is very chaotic for the older patient with multiple health problems. There are often multiple consultations and a transfer to a new ward. This can lead to confusion for the patient and their family

The care on regular wards in the Netherlands is very different, as visualized in Fig. 2. There is a lot of separate deliberation between residents, supervisors, and consultants making it a less efficient process. Because of multiple consultations, the patient sees multiple doctors by their bed which can be confusing. In addition, residents may alternate between departments on a day to day basis, causing the patient to see even more different faces during their admission, which can add to the confusion. Patients often need to be transferred to a different ward, meaning they are placed in a completely new environment which can further increase confusion for the patient.

### Study population and setting

The study was conducted at the Jeroen Bosch Hospital, a large teaching hospital in the Netherlands, where the ICW has been operating since 15 June 2020. The ICW group consisted of patients admitted to the ICW between 15 June 2020 and 31 October 2020, with the indication for the ICW being determined by the main treating specialist in the ER. The indication for ICW admission is a combination of health problems covered by the specialties involved and/or uncertainty as to which specialty should be responsible, e.g. dyspnea of unknown origin, and indication for hospital admission.

The control group consisted of a historical cohort of comparable patients treated in regular wards in 2019, as there was no ICW in 2019. Selection was as follow: patients presenting between 15 June 2019 and 31 October 2019 to the ER were retrospectively screened for an ICW indication, to determine if they would have been admitted to the ICW if there had been one in 2019. This was determined by a specialist (cardiologist, internist, geriatrician, or pulmonologist) based on the ER correspondence, to mimic the similar procedure followed for ICW admission. The specialist were asked: "Would you or would you not admit the patient to the ICW based on the emergency department's conclusions?" without knowing the patient's outcome. Exclusion criteria for both groups were: 1) patients admitted through an outpatient clinic and, 2) patients who had to be transferred to a coronary care unit or intensive care unit during admission, as patient outcomes could no longer be influenced by the collaborative practice being studied.

### Data collection

Data were retrospectively extracted from the patients’ electronic medical records, using the data mining software system CTcue (CTcue BV, Amsterdam, https://ctcue.com/) and the in-hospital health information management department. All data was electronically retrieved except for the medical history, this was manually retrieved from the letter from the ER visit.

### Variables

Baseline variables were age, sex, medical history, number of medications used at the time of ER visit, number of hospital admissions in the last 12 months, and admission specialty.

Outcomes were the number of medical consultations in the ER (both calls and in-person visits), medical in-hospital consultations, allied health professional consultations, number of radiological procedures, waiting time for radiological procedures, change in primary treating specialty, length of hospital stay, readmission rates, and mortality rates. A medical consultation is defined as a doctor who visits or is called about the patient for examination to provide advice about the diagnosis or treatment at the request of the primary treating specialist. An allied health professional consultation on the other hand, is defined as an allied health professional visiting a patient on the ward to provide health-promoting or supportive services at the request of the primary treating specialist. The included allied health professionals were physical therapist, dietitian, speech therapist, occupational therapist, and liaison nurse. The number of consultations in the ER and in-hospital, the number of allied health professional consultations, and radiological procedures were presented in two ways. First, as the average number of consultations or procedures per patient because of its clinical relevance and for the sake of readability. Second, the most methodologically correct presentation, as the data are highly skewed and this ordinal presentation also allows for the correction of confounders. The categories were as follows: 0, 1, 2 and ≥ 3 for specialists consultations, and 0, 1, 2, 3 and ≥ 4 for allied health professionals. Readmission rates were cumulatively evaluated for 30 days, 3 months, and 12 months after the primary admission. Mortality rates were cumulatively evaluated for in-hospital deaths, and after 30 days, 3 months, and 12 months.

### Statistical analysis

Continuous baseline variables were evaluated for normality distribution. The variable “medications at admission” was normally distributed and was evaluated using an independent sample t-test. The variable “age” was skewed and contained outliers, and was therefore evaluated using Mood’s median test since this test is more robust against outliers than the Mann–Whitney U test. All other baseline variables were evaluated using a Chi-Square test. Baseline differences between groups were added as covariates to the main analysis to adjust for potential confounding.

**Fig. 3:**
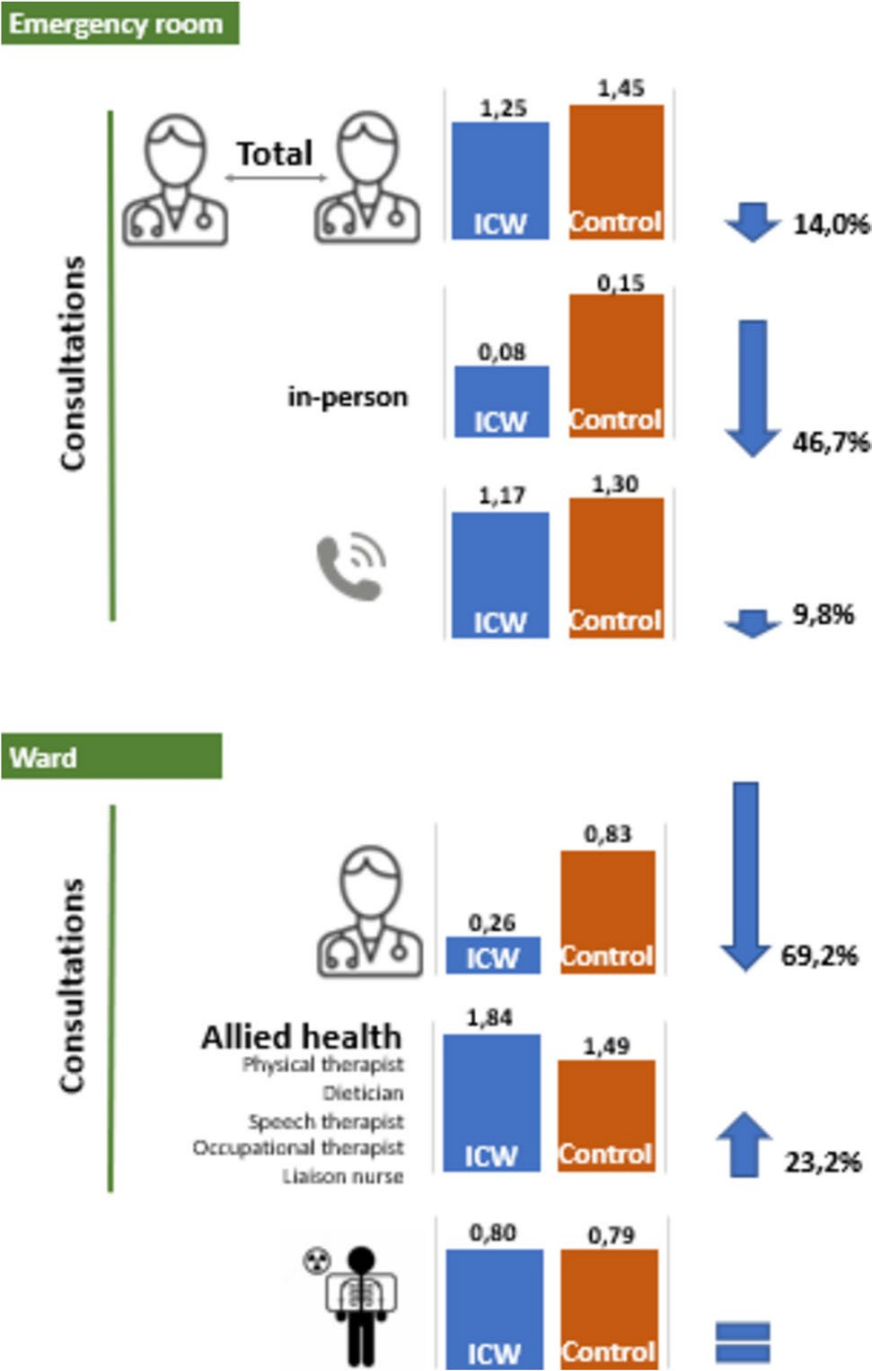
Results of ICW care: clinical relevance An arrow indicates a relevant difference. An “ = ” indicates there is no relevant difference

First the outcomes ER, in-hospital and allied health consultations, and the number of radiological procedures were presented descriptively (Fig. 3). Second all outcomes were analyzed by either linear, logistic binary, or logistic multinomial regression models, where appropriate. All regression analyses were carried out with adjustment for baseline differences. All analyses were carried out using SPSS (IBM SPSS Statistics for Windows, Version 25.0. Released 2017. Armonk, NY: IBM Corp), with two-sided *p*-value < 0.05 denoting statistical significance.

In addition, we conducted a sensitivity analysis for the length of hospital stay, since we expected this to be affected by two external factors. One factor is the waiting time for post-hospital rehabilitation, which may have differed between the control group and the ICW group because of the COVID-19 pandemic in 2020 which may affect the availability of rehabilitation facilities. Patients were considered “waiting” if they were discharged to an institution they had not been staying previously, as this may give rise to a waiting period. For example, a patient who has been living at home but has been discharged to a nursing home after a hospital stay may have to wait for a bed to become available. Another factor is the shared decision to start providing palliative care, which may either prolong or reduce the length of hospital stay in either study group. For the sensitivity analysis, patients who had to wait for post-hospital care or who received palliative care were excluded, and group differences in length of hospital stay were again analyzed using linear regression.

## Results

### Patient characteristics

A total of 200 ICW and 239 control patients were included in the study. There were six patients who were in both the ICW and in the control group. Patient characteristics were similar in both groups, except for the distribution in admission specialty (Table 1). Age was bordering statistically different (p = 0.052) and was identified as a potential confounder. Both admission specialty and age influenced the crude outcome > 10% and therefore outcomes were adjusted for both.

**Table 1:** Baseline characteristics of patients in the Intensive Collaboration Ward (ICW) And Control Group

	**ICW** *n* = 200	**Control** *n* = 239	
	**Descriptives**		**Statistics**
	**n (%)**	**n (%)**	***p*-value**
**Age median (IQR)**^a^	81.5 (14)	79 (17)	0.052
**Female**^b^	105 (52.5)	115 (48.1)	0.360
**Admission specialty**^b^			< 0.001*
Internal medicine	64 (32.0)	108 (45.2)	
Pulmonary medicine	51 (25.5)	79 (33.1)	
Geriatric medicine	73 (36.5)	42 (17.6)	
Cardiology	12 (6.0)	10 (4.2)	
**Medications at admission mean (SD)**^c^	9.2 (5.0)	8.4 (4.6)	0.099
**Admissions past 12 months**^b^			0.750
0	123 (61.5)	142 (59.4)	
1	41 (20.5)	54 (22.6)	
2	18 (9.0)	22 (9.2)	
3	5 (2.5)	10 (4.2)	
≥ 4	13 (6.5)	11 (4.6)	
**Medical history**			
Internal medicine ^b^	117 (58.5)	141 (59.0)	0.916
Diabetes mellitus	57 (28.5)	62 (25.9)	0.548
Hematological disease	6 (3.0)	12 (5.0)	0.288
Kidney disease	32 (16.0)	34 (14.2)	0.604
Auto-immune disease	1 (0.5)	7 (2.9)	-
Other	66 (33.0)	81 (33.9)	0.844
Pulmonary medicine ^b^	100 (50.0)	133 (55.6)	0.238
COPD/asthma	57 (28.5)	76 (31.8)	0.454
Malignancy	6 (3.0)	17 (7.1)	0.054
Other	64 (32.0)	94 (39.3)	0.111
Geriatric medicine ^b^	70 (35.0)	75 (31.4)	0.422
Cognitive/neurodegenerative	24 (12.0)	30 (12.6)	0.861
CVA	46 (23.0)	48 (20.1)	0.458
Hip fracture	10 (5.0)	6 (2.5)	0.166
Other	1 (0.5)	1 (0.4)	-
Cardiology ^b^	150 (75.0)	174 (72.8)	0.602
ACS	58 (29.0)	63 (26.4)	0.538
Heart failure	35 (17.5)	42 (17.6)	0.984
AP stable	13 (6.5)	13 (5.4)	0.630
Artery disease	30 (15.)	31 (13.0)	0.540
CVRM	89 (44.5)	96 (40.2)	0.360
Other	74 (37.0)	84 (35.1)	0.687

- The expected count in the Chi-square test was too low to interpret the *p*-value

*COPD* Chronic obstructive pulmonary disease, *CVA* Cerebrovascular accident, *ACS* Acute coronary syndrome, *AP* Angina pectoris, *CVRM* Cardiovascular risk management

^*^Significant difference *p* < 0.05

^a^Median test for k samples

^b^Chi-square test

^c^Independent sample t-test

### Main results

Descriptive analysis showed that in the emergency room ICW patients required less consultations from other specialties than the control patients (-14%), both in person (-47%) as per phone (-10%) (Fig. 3). When admitted to the ward, this difference is even larger: a decrease from an average of 0.83 consultations per patients to 0.26 per patient on the ICW (-69%). We saw an increase in number of consultations by allied health professionals on the ICW (+ 23%). The average number of radiological tests per patient did not change.

A more in dept analysis of the outcomes, adjusted for baseline differences, showed similar findings: ICW patients required significant fewer ER consultations than the control group: 25.0% and 37.3% of patients, respectively, had two or more ER consultations (Table 2). The in-person consultations did decrease, but did not reach statistical significance. In both groups, at least one call was made to a specialist for most patients, these calls often being made by residents to their supervisors. However, a second call to a specialist was required less often for patients in the ICW group than for patients in the control group: 15.5% versus 24.7%, a significant decrease compared to the control group (OR 0.14, CI 0.03–0.54). ICW patients required significantly fewer in-hospital consultations in each category (1, 2, or ≥ 3) than control patients (respective ORs 0.34 (CI 0.21–0.55), 0.11 (CI 0.04–0.29), and 0.07 (CI 0.02–0.33)).

**Table 2:** Outcomes of patients in the intensive collaboration ward (icw) compared to the control group

	**ICW** *n* = 200	**Control** *n* = 239	**Adjusted for baseline differences**	
			**OR**	**95% CI**
**Number of emergency room consultations**^a^				
0	10 (5.0)	2 (0.8)	*Reference category*	
1	140 (70.0)	148 (61.9)	0.16*	0.03–0.74
2	43 (21.5)	74 (31.0)	0.10*	0.02–0.50
≥ 3	7 (3.5)	15 (6.3)	0.08*	0.01–0.47
**Of which in person consultations**^**b**^				
0	185 (92.5)	208 (87.0)	*Reference category*	
≥ 1	15 (7.5)	31 (13.0)	0.53	0.27–1.02
**Of which calls to specialists**^a^				
0	10 (5.0)	3 (1.3)	*Reference category*	
1	153 (76.5)	170 (71.1)	0.22*	0.06–0.83
2	31 (15.5)	59 (24.7)	0.14*	0.03–0.54
≥ 3	6 (3.0)	7 (2.9)	0.23	0.04–1.27
**Number of in-hospital consultations**^**a**^				
0	158 (79.0)	118 (49.4)	*Reference category*	
1	35 (17.5)	72 (30.1)	0.34*	0.21–0.55
2	5 (2.5)	31 (13.0)	0.11*	0.04–0.29
≥ 3	2 (1.0)	18 (7.5)	0.07*	0.02–0.33
**Number of allied health professional consultations**^**a**^				
0	51 (25.5)	62 (25.9)	*Reference category*	
1	41 (20.5)	84 (35.1)	0.53*	0.30–0.91
2	43 (21.5)	39 (16.3)	1.21	0.67–2.19
3	27 (13.5)	34 (14.2)	0.83	0.43–1.60
≥ 4	38 (19.0)	20 (8.3)	2.03*	1.02–4.04
**Number of radiological procedures**^**a**^				
0	112 (56.0)	150 (62.8)	*Reference category*	
1	49 (24.5)	41 (17.2)	1.38	0.84–2.26
2	21 (10.5)	22 (9.2)	1.19	0.61–2.30
≥ 3	18 (9.0)	26 (10.9)	0.95	0.49–1.83
**Change in primary treating specialty**^b^				
	31 (15.5)	11 (4.6)	4.50*	2.16–9.40
**Readmission rate**^**a**^ (cumulative)				
30-day	27 (13.5)	25 (10.5)	1.47	0.81–2.66
3-month	42 (21.0)	55 (23.0)	0.93	0.58–1.47
12-month	72 (36.0)	83 (34.7)	1.06	0.71–1.58
**Mortality rate**^**a**^ (cumulative)				
In hospital	17 (8.5)	24 (10.0)	0.73	0.37–1.45
30-day	35 (17.5)	36 (15.1)	1.04	0.61–1.79
3-month	52 (26.0)	47 (19.7)	1.28	0.80–2.06
12-month	80 (40.0)	77 (32.2)	1.22	0.80–1.85
	**median (IQR)**	**median (IQR)**	**Β**	**95% CI (B)**
**Waiting time for radiological procedures in hours**^**c**^				
	5 (19)	3 (20)	0.09	-1.45–9.28
**Length of hospital stay in days**^**c**^				
	5 (5)	5 (5)	-0.02	-1.36–0.83

^*^ Significant difference *p* < 0.05

^a^ Multinomial logistic regression

^b^ Binary logistic regression

^c^ Linear regression

ICW patients significantly less often had 1 allied health professional consultation (OR 0.53, CI 0.30–0.91), but significantly more often had 4 or more consultations (OR 2.03, CI 1.02–4.04). The primary treating specialty was changed significantly more often among ICW patients than among control patients (15.5% vs 4.6%, respectively; OR 4.50, CI 2.16–9.40).

Length of hospital stay, readmission and mortality rates, and the number of and waiting time for radiological procedures did not differ statistically significant between the two groups.

### Sensitivity analysis

Significantly more ICW patients (15.5%) than control patients (9.2%) had to wait for post-hospital rehabilitation or care. Palliative care was started in a similar proportion of patients in the two groups (ICW 6.0% and control 6.7%). After exclusion of these patients, we re-evaluated a total of 157 ICW and 202 control patients in the sensitivity analysis. Length of hospital stay was reduced to a median of 4 days in both groups, which was not significantly different.

## Discussion

This study demonstrated that providing care centered around a multimorbid patient on an ICW resulted in a clinically relevant and statistically significant decrease in consultations, compared to standard monodisciplinary care. Fewer medical consultations were needed for ICW patients in the ER and also while in the ward. ICW patients were seen more often by allied health professionals. ICW patients primary treating specialty was changed more often, but this does not lead to changing of a ward as it is centered in the ICW. There were no differences in the number of, and waiting time for, radiological procedures, length of hospital stay, readmission rates, and mortality rates.

We concluded that patients in the ICW group required significantly fewer in-hospital consultations than the patients in the control group (no consultation in 79.0% and 49.4%, respectively). Previous studies and systematic reviews done by Reeves, Gougeon, Pannick, Shakib, and Puelle did not report on the number of consultations with medical specialists other than those involved in the collaboration [12–14, 21, 22]. The results of our study suggest that care was less fragmented in the ICW group than in the control group. In addition, patients in the ICW group required significantly fewer ER consultations, mainly due to a reduction in the number of consultations with specialists other than the patient's own consultant. This may be clinically relevant when taking into account the effect of being disturbed during other duties, which is the case with unscheduled consultation requests. Research shows that being disturbed increases the likelihood of errors being made [23, 24] and it takes a person at least 15 min to re-concentrate on what they were doing before being disturbed [25, 26]. The daily scheduled treatment team meetings are probably the reason for the decrease in consultations when admitted to the ICW. ER consultations probably decreased because a patient does not have to be admitted to a specific specialty ward and thus does not require consultations by different specialties to decide where a patient should be admitted. The difference in in-person consultations in the ER was not significant, which is most probably due to the low incidence of in-person consultations (7.5% in the ICW and 13.0% in the control group). It is difficult to compare our data with those of other studies because of the heterogeneity of studies [12, 13]. The cohort study by Puelle et al. found that interprofessional collaboration between geriatricians and a hospitalist increased geriatric consultations by 2.3absolute percentage points [22]. However, the aim of the intervention was to increase geriatric consultations and the authors did not report on medical consultations outside of their collaboration, whereas we focused on all consultations.

Patients in the ICW group were seen significantly more often by allied health professionals than patients in the control group (an average of 1.84 versus 1.49 involved professionals per patient). This could be explained by the collaborative practice with frequent interprofessional and intraprofessional evaluation of the patient, resulting in more attention for the entirety of a patient’s health problems and wellbeing, which is in line with the concept of positive health [27]. Allied health professionals provide a wide range of services to help patients achieve optimal wellbeing, in addition to implementing treatment prescribed by medical specialists. To the best of our knowledge, we are the first to report allied health professional consultations as an outcome instead as a part of the intervention. Other studies did not focus on the number of allied health professional consultations [12–14, 21, 22]. Allied health professional consultations were not standard for all patients admitted to the ICW, but were implemented based on the needs of the patient and were thus a result of patient-centered care.

The primary treating specialty was changed more often on the ICW. This is probably because it is difficult to establish the main problem in patients with multiple health disorders. We suggest that the intraprofessional patient meetings on the ICW helped clarify the situation, often leading to a change in the primary treating specialty. This, in turn, may have also contributed to the decrease in medical consultations, if similar control patients were admitted to the “wrong” specialty ward and needed to be seen by different medical specialists to establish the primary health problem. The ICW appears to provide the right care in the right place, with clear communication from one doctor, as shown in Figs. 1 and 2. This can be seen as a better quality of care. Also, for the ICW group, a change in primary specialty does not result in the patient being moved and having to adjust to a new ward. This makes it logistically easier for the ICW group to change their primary specialty without any negative impact on the patient.

We found no significant difference in length of stay (LOS). Previous studies have also reported on the LOS when interprofessional collaboration is implemented. Reeves et al. reported one study with a reduced LOS of 0.6 days, but also one study with no difference in LOS. Gougeon et al. and Shakib et al. also found no difference in the LOS. Pannick et al. found that 70% of the interprofessional interventions studied did not improve the length of stay, and those that did reduced the length of stay by less than 0.5 days. However, in an earlier study, the ICW was found to reduce the length of hospital stay by two days [16]. We carried out a sensitivity analysis for two factors that are known to influence hospital stay: waiting time for post-hospital rehabilitation or care and the shared decision to start providing palliative care. Although significantly more ICW patients had to wait for post-hospital rehabilitation or care, probably because of shortage of appropriate beds in 2020 because of the SARS-CoV-19 pandemic, there was still no significant difference between the ICW and the control group: the length of hospital stay was reduced in both groups to a median of 4 days. A possible explanation for the lack of difference in the length of stay may be because we studied two different time periods whereas the previous study compared groups in the same time period, thereby eliminating *all*factors that influence the length of stay. The same time period method is the preferred study design for the length of hospital stay, such as a previous study on the ICW in which a within time period analyses did show a decrease in length of stay namely from median 7 days to median 5 days [16].

This study had some limitations. First, patients that were part of the control group were admitted a year prior to the opening of the ICW, which makes comparisons difficult because of potential differences such as waiting time for post-hospital care, as described above. On the other hand, this design eliminated the risk of ‘contamination of knowledge’ which occurs when comparing groups within one time period. In a within one time period design, specialists can gain knowledge from the ICW collaboration and apply it in the regular care ward, which generates contamination of knowledge and can influence outcomes such as the number of consultations. In this between time period design this is not possible. Second, there was a significant difference in the baseline variable ‘admission specialty’, the results were adjusted for this accordingly. Third, it is possible that the knowledge of staff working on the ICW and the degree of collaboration increased over time, which may have led to an underestimation of the effect of the interprofessional and intraprofessional care in the ICW group. Fourth, some patients were included in the intervention group and control group, but a sub-analysis of this group was not possible due to the limited number of these patients. However, because of the limited number, we would not expect them to have a significant effect on study outcomes. Lastly, the control group was selected based on the ER letter by a single specialist of the corresponding specialty, so in total four specialists included patients. This might generate selection bias since the specialists screened the ER letters with the study aim in mind. However, the specialists were provided with the least possible information to prevent bias. They were asked: “if there was a ICW in 2019, would you admit this patient to the ICW or not, based on the ER conclusions for each patient”. In addition, they did not have any insight into the patients’ outcomes, and were not involved in the data collection or analysis. The involved specialists were involved in the data interpretation and writing of the manuscript.

## Conclusions

While recognizing the limitations of our study, and adjusting for them where possible, we can conclude that the interprofessional and intraprofessional collaborative practice on the ICW reduced the number of medical consultations needed, which might be an important sign of defragmentation of care and more integrated and efficient care. Combining these between-time period results with the results of the within one time period study of De Gans et al., [16] we believe the ICW has a clinically relevant positive effect on the efficiency of care and patient-centered care. It would be interesting to study the experiences and opinions of patients and healthcare providers about the care provided on the ICW. Further research is required to evaluate interprofessional and intraprofessional collaboration in terms of the quadruple-aim: improved health outcomes, enhanced patient experience, improved work life of healthcare providers, and lower costs [28].

## Data Availability

The datasets used and analyzed during the current study are available from the corresponding author on reasonable request.

## Abbreviations

ICW: Intensive Collaboration Ward
ER: Emergency Room
TTM: Treatment Team Meeting

## References

[1] Murray CJL, Barber RM, Foreman KJ (2015). Global, regional, and national disability-adjusted life years (DALYs) for 306 diseases and injuries and healthy life expectancy (HALE) for 188 countries, 1990–2013: quantifying the epidemiological transition. Lancet.

[2] World Health Organization. The economics of healthy and active ageing series – Living longer, but in better or worse health? [Internet] Available from: https://apps.who.int/iris/bitstream/handle/10665/332075/Policy-brief-1997-8073-2020-1-eng.pdf?sequence=11&isAllowed=y. Accessed 4th March 2022.

[3] Hopman P, Heins MJ, Korevaar JC (2016). Health care utilization of patients with multiple chronic diseases in the Netherlands: differences and underlying factors. Eur J Intern Med.

[4] Soley-Bori M, Ashworth M, Bisquera A (2020). Impact of multimorbidity on healthcare costs and utilisation: a systematic review of the UK literature. Br J Gen Pract.

[5] Lehnert T, Heider D, Leicht H (2011). Review: health care utilization and costs of elderly persons with multiple chronic conditions. Med Care Res Rev.

[6] World Health Organization. Multimorbidity – Technical Series on Safer Primary Care. Available from:

[7] Liu CW, Einstadter D, Cebul RD (2010). Care fragmentation and emergency department use among complex patients with diabetes. Am J Manag Care.

[8] Frandsen BR, Joynt KE, Rebitzer JB, Jha AK (2015). Care fragmentation, quality, and costs among chronically ill patients. Am J Manag Care.

[9] World Health Organization. Framework For Action On Interprofessional Education & Collaborative Practice. Available from: https://apps.who.int/iris/bitstream/handle/10665/70185/WHO_HRH_HPN_10.3_eng.pdf?sequence=1. Accessed 4th March 2022.

[10] Taylor C, Munro AJ, Glynne-Jones R (2010). Multidisciplinary team working in cancer: what is the evidence?. BMJ.

[11] Whitty CJM, MacEwen C, Goddard A (2020). Rising to the challenge of multimorbidity. BMJ.

[12] Reeves S, Pelone F, Harrison  R (2017). Interprofessional collaboration to improve professional practice and healthcare outcomes. Cochrane Database Syst Rev.

[13] Gougeon L, Johnson J, Morse H (2017). Interprofessional collaboration in health care teams for the maintenance of community-dwelling seniors' health and well-being in Canada: A systematic review of trials. J Interprof Educ Pract.

[14] Pannick S, Davis R, Ashrafian H (2015). Effects of Interdisciplinary Team Care Interventions on General Medical Wards: A Systematic Review. JAMA Intern Med.

[15] Kammerlander C, Roth T, Friedman SM (2010). Ortho-geriatric service—a literature review comparing different models. Osteoporos Int.

[16] de Gans S, Penturij-Kloks M, Scheele F, van de Pol M, van der Zwaard B, Keijsers C (2022). Combined interprofessional and intraprofessional clinical collaboration reduces length of stay and consultations: a retrospective cohort study on an intensive collaboration ward (ICW). J Interprof Care.

[17] Mainous AG, Baker R, Parker SG (2000). Hospitalists for the NHS?. J R Soc Med.

[18] Ziekenhuisarts. KNMG. Retrieved 3 June 2022, from https://www.knmg.nl/opleiding-herregistratie-carriere/geneeskundestudie/overzicht-opleidingen-1/ziekenhuisarts.htm.

[19] Mitzkat A, Berger S, Reeves S, Mahler C. (2016). More terminological clarity in the interprofessional field - a call for reflection on the use of terminologies, in both practice and research, on a national and international level. GMS Journal For Medical Education, 33(2). 10.3205/zma001035. 10.3205/zma001035 PMC4895843 27280147

[20] Reinders JJ, Pesut D. (2022). A Meta-Model for Transforming Interprofessional Practice, Education, and Research. In Interprofessional Education and Collaborative Practice: International Approaches at the Micro, Meso, and Macro Levels. Cognella Academic Publishing.

[21] Shakib S, Dundon BK, Maddison J (2016). Effect of a Multidisciplinary Outpatient Model of Care on Health Outcomes in Older Patients with Multimorbidity: A Retrospective Case Control Study. PLoS ONE.

[22] Puelle M, Wiggins J, Khateeb R (2018). Interprofessional Intervention to Improve Geriatric Consultation Timing on an Acute Medical Service. J Am Geriatr Soc.

[23] Li SYW, Blandford A, Cairns P, Young RM (2008). The effect of interruptions on postcompletion and other procedural errors: an account based on the activation-based goal memory model. J Exp Psychol Appl.

[24] Feuerbacher RL, Funk KH, Spight DH (2012). Realistic distractions and interruptions that impair simulated surgical performance by novice surgeons. Arch Surg.

[25] DeMarco T, Lister T (1987). Peopleware: Productive Projects and Teams.

[26] Jackson T, Dawson R, Wilson D (2003). Reducing the effect of email interruptions on employees. Int J Inf Manage.

[27] Huber M, van Vliet M, Giezenberg M (2016). Towards a ‘patient-centred’ operationalisation of the new dynamic concept of health: a mixed methods study. BMJ Open.

[28] Bodenheimer T, Sinsky C. From triple to quadruple aim: care of the patient requires care of the provider. Ann Fam Med. 2014;12(6):573–576. 10.1370/afm.1713 PMC4226781 25384822

